# Jianpi Jieyu Decoction, An Empirical Herbal Formula, Exerts Psychotropic Effects in Association With Modulation of Gut Microbial Diversity and GABA Activity

**DOI:** 10.3389/fphar.2021.645638

**Published:** 2021-04-14

**Authors:** Lanying Liu, Zhilu Zou, Jiangwei Yang, Xiaoqi Li, Boran Zhu, Hailou Zhang, Yan Sun, Yuxuan Zhang, Zhang-Jin Zhang, Wei Wang

**Affiliations:** ^1^ Department of Psychiatry, Tongde Hospital of Zhejiang Province, Hangzhou, China; ^2^ Mental Health Center of Zhejiang Province, Hangzhou, China; ^3^ College of Basic Medicine, Hubei University of Chinese Medicine, Wuhan, China; ^4^ Key Laboratory of Integrative Biomedicine of Brain Diseases, Nanjing University of Chinese Medicine, Nanjing, China; ^5^ School of Chinese Medicine, LKS Faculty of Medicine, The University of Hong Kong, Hong Kong, China

**Keywords:** depression, Jianpi Jieyu decoction, gut microbiota, *Lactobacillus*, GABA

## Abstract

**Background:** Recent studies suggest that gut microbiota was associated with the bidirectional gut-brain axis which could modulate neuropsychological functions of the central nervous system. Gut microbiota could produce gamma aminobutyric acid (GABA) that could modulate the gut–brain axis response*.* Jianpi Jieyu (JPJY) decoction, a traditional Chinese formula, is mainly composed of *Astragalus membranaxeus* and Radix *Pseudostellariae*. Although the JPJY decoction has been used to treat the depression in China, the potential action of its antidepressant has not been well understood. Thus this study was aim to investigate the role of JPJY improve gut microbiota homeostasis in the chronic stress induced depressive mice.

**Methods:** The antidepressant effect of JPJY on chronic unpredictable mild stress (CUMS) mice was evaluated by using sucrose preference test, tail suspension test and forced swim test. Fatigue-like behaviors were evaluated using degree of redness, grip strength test, and exhaustive swimming test. The new object recognition test was used to evaluate cognition performance. Fecal samples were collected and taxonomical analysis of intestinal microbial distribution was conducted with 16S rDNA. Serum level of GABA was measured using high performance liquid chromatography (HPLC). The expression of GluR1 and p-Tau protein in the hippocampus was determined using Western blotting.

**Results:** The dose of 9.2 g/kg JPJY produced antidepressant-like effects. JPJY and its major components also modulated gut microbiota diversity in the CUMS mice. Serum level of GABA and the expressions of hippocampal GluR1 and p-Tau were reversed after the administration of JPJY in CUMS mice.

**Conclusion:** JPJY regulates gut microbiota to produce antidepressant-like effect and improve cognition deficit in depressive mice while its molecular mechanism possibly be enhanced NR1 and Tau expression in hippocampus and increased GABA in serum.

## Introduction

Major depressive disorder (MDD) is severe and persistent emotional depression, which is the main type of mood disorder and has caused an enormous socioeconomic burden (Chen and Guo, 2017; Woelfer et al., 2019). Herein, it is urgently to be solved in the worldwide. Fatigue is also a common health problem with a high prevalence. In clinic, the depressive patients are often accompanied with fatigue symptoms which can negatively affect daily functioning (Lam et al., 2012). Studies have shown that the depression and fatigue shared a partial genetic predisposition, which may result in a co-susceptibility to depression and fatigue (Felger et al., 2012).

Studies indicated that the gut microbiota maintains a dynamic balance which plays an important role in human health through regulating nutrient absorption, immune system and host metabolism (Cryan and Dinan, 2012; Browne et al., 2016; Fung et al., 2017). It has been reported that the composition of gut microbiota was closely related to the mental disease, such as depression, bipolar and autism. (Zheng et al., 2016). Compared the fecal microbiota of depressed patients with normal people, it was found that the cecal gut microbiota of depressed patients mainly manifested by the high proportion of *Bacteroides,* and was obvious shortage of *Lachnospiraceae* (Koren et al., 2012). Different *lactobacillus* and *Bifidobacteria* species have been reported to regulate depression and stress-related behaviors in animal rodents (Savignac et al., 2015; Kelly et al., 2016). Thus, the gut microbiota may play a causal role in the development of characteristics of depression, and it may provide a manageable target in the treating and preventing this disease.

Gamma aminobutyric acid (GABA) is the principal inhibitory neurotransmitter playing a key role in anxiety and depression disorders in mammals (Dhakal et al., 2012; Sarasa et al., 2020). There is a growing evidence that GABA dysfunction is one of the culprits in depression, where levels of GABA are low in the cerebrospinal fluid (Kali, 2016). Recent studies revealed that the members of gut microbiota were able to produce gamma aminobutyric acid (GABA) which could modulate the gut–brain axis response, such as *Bifidobacterium adolescentis* (Barrett et al., 2012; Duranti et al., 2020). In germ-free mice, it has been reported that GABA levels in feces and blood were significantly reduced (Matsumoto et al., 2017; Strandwitz et al., 2019). Similarly, in certain pathogen-free mice, GABA levels in feces can be alteredby antibiotics, suggesting that the microbiota may contribute to circulating levels of GABA (Fujisaka et al., 2018).

Jianpi Jieyu decoction (JPJY) is composed as follows: *Astragali radix* (Huang Qi), *Radix Pseudostellariae* (Tai Zi Shen), *Radix Et Caulis Acanthopanacis Senticosi* (Ci Wu Jia), *Rhizoma Atractylodes Macrocephalae* (Bai Zhu), *Radix Albus Paeoniae Lactiflorae* (Bai Shao), *Concha Margaratiferae* (Zhen Zhu Mu), *Radix Polygalae* (Yuan Zhi), and *Radix Glycyrrhizae* (Gan Cao). *Astragali radix* has been proven to ameliorate fatigue-like behaviors both in human and rats (Kuo et al., 2009; Zhou et al., 2017). Studies also have found that *Astragali radix* can improve depressing-like behavior, reverse the memory impairment and neurodegeneration (Liu et al., 2017; Song et al., 2018). *Radix Pseudostellariae* has significant activities such as antitumor, immunomodulatory activities and ameliorating chronic fatigue syndrome, contributing to its abundant bioactive compounds like polysaccharides. (Sheng et al., 2011). The drug pair of *Radix Bupleuri* and *Radix Albus Paeoniae Lactiflorae* had an excellent antidepressant effect (Wang et al., 2019). *Radix Polygalae* displayed the antidepressant effect in chronic unpredictable mild stress mice *via* inhibiting NF-κB regulation which depends on NLRP3 signaling pathway (Li et al., 2017), and it also has neuroprotective and neurodegenerative effects (Yu et al., 2014). Although the JPJY decoction has been used to treat the depression in China, it was limited to the potential action of its antidepressant. Therefore, in this study, we aimed to determine the effect of administration with JPJY decoction on the regulation of gut microbiota and reduction of depressive symptoms in mice.

## Materials and Methods

### Animals

Male Balb/c mice were obtained from the Changzhou Kavins Experimental Animal Co. LTD. (license number: SCXX 2017-0001). Mice aged approximately 6 weeks old (20 ± 2 g). The animals were kept in the Animal Center of Nanjing University of Chinese Medicine at room temperature of 25 ± 2°C, with 12 h light and dark cycle. Excluding the experimental time, mice were fed freely with food and water. Before the behavioral test, the mice were used to the animal facility for 7 days. All animal experiments were in accordance with the Guide for the Care and Use of Laboratory Animals approved by the Institutional Animal Care and Use Committee at Nanjing University of Chinese medicine.

### Drugs

Jianpi Jieyu decoction is composed as follows: *Astragalus mongholicus Bunge* (Huang Qi, 20 g), *Pseudostellaria heterophylla* (Miq.) *Pax* (Tai Zi Shen, 12 g), *Eleutherococcus senticosus* (Rupr. and Maxim.) *Maxim* (Ci Wu Jia, 15 g), *Atractylodes macrocephala Koidz* (Bai Zhu, 12 g), *Paeonia lactiflora Pall* (Bai Shao, 15 g), *Concha Margaratiferae* (Zhen Zhu Mu, 30 g), *Polygala tenuifolia Willd* (Yuan Zhi, 12 g), and *Glycyrrhiza glabra* L (Gan Cao, 9 g). These materials were provided by the pharmacy of Zhejiang Tongde Hospital. Zhen Zhu mu was firstly soaked with water for 30 min at a solid liquid ratio of 1/6 (g/ml) and then extracted under reflux for 30 min. The rest of materials were soaked with water for 30 min at a solid liquid ratio of 1:8 (g/ml), and were then extracted two times (a solid liquid ratio of 1/8, 1/6, respectively) under reflux for 1 h. All the supernates were mixed, and the solvents were then removed by using a rotary evaporator at 60°C to obtain JPJY extracts. The extracts were stored at −20°C for the future study. Huangqi-Taizishen (QS) were extracted as above method. Fluoxetine hydrochloride was purchased from Sigma-Aldrich, and was dissolved with 0.9% saline to prepare a solution of 18 mg/kg before being used for intraperitoneal injection. JPJY and QS were administrated by using intragastric gavage, and fluoxetine was administrated by using intraperitoneal injection.

### Chronic Unpredictable Mild Stress (CUMS)

The CUMS modeling process was slightly modified as previously described method (Tang et al., 2015). The mice were placed individually and subjected to mild stress for 4 weeks. The stresses were as follows: damp cage for 16 h (the mice were placed in a mouse cage with damp padding), water deficiency for 24 h, fasted for 24 h, 45 cage tilt for 24 h, 200 r/min shaking for 40 min, bound for 6 h (the mice were bound in a 50 ml self-made tethered tube for 6 h, the mice were restricted in activity), twice a week lightening throughout the night (100 W light for 12 h). The order in which the mice were subjected to stress per day changed every week except that the nighttime illumination was unchanged twice a week, per every 3–4 days.

After finishing modeling in the 4th week, the model group was randomly divided into four groups: Veh group (saline), JPJY group (9.2 g/kg JPJY), QS group (6.9 g/kg QS), mFlx group (20 mg/kg of Flx).

After administration for 2 weeks, the behavioral tests were tested as follows: opening field test, grip strength, degree of redness, tail suspension, and exhausted swim test. There is at least an interval of 2 h between the two tests. The next day, sucrose preference, novelty-suppressed feeding test, and forced swimming were tested.

### Behavioral Tests

All behavioral tests were carried out in the late light phase. Animals were transferred to the laboratory and used to the room conditions for at least 1 h before the beginning of the behavioral experiments. Behavioral testers were blinded to the experimental groups.

#### Open Field Test (OFT)

The mice were placed alone in an open field of 40 cm × 40 cm × 40 cm and explored freely. A camera was installed above the open box for to record locomotor activity. Mice were placed in the center of the arena, allowing free exploration for 5 min. The total distance traveled and time spent in central area were measured. Before introducing each animal, thoroughly clean the test instrument with 70% ethanol.

#### Tail Suspension Test (TST)

The instrument consists of six chambers. The front of the box was open, with a horizontal stick 1 cm from the top and a vertical stick suspended in the middle. Mice were individually suspended 1 cm from the tip of the tail to the vertical bar with adhesive tape. A camera placed in front of the TST box recorded the animals’ behavior for 6 min. The software analyzed the Immobility time of the last 4 min. Mice were returned to individual cages and remained so until the end of the experiment.

#### Forced Swimming Test (FST)

The mice were placed in a transparent plexiglass (25 cm high; 10 cm in diameter) filled with a depth of 10 cm water (23–26°C). A camera recorded the 6 min swimming session. The immobility of the mice was measured during the last 4 min of the experiment. In this test, immobility was defined as their passive floating in the water.

#### Novelty-Suppressed Feeding Test (NSF)

After fasting for 18 h, the mice were conditioned for 2 h in the experimental environment and then they were introduced to a new cage with weighed grain placed in the center. Each mouse was placed in a corner of the box and allowed to explore up to 10 min. When the mice chewed part of the food, the experiment was over. The time mouse began to eat the food pellet was regarded as the latency. At the end of the test, mice returned to their home cage, and food consumption was quantified for 10 min to verify the absence of differences in hunger and/or motivation. The amount of food consumed in the original cage is based on the weight of mice food eaten within in total 10 min. Food consumption is the weight of chow consumed divided by the weight of the mice.

#### Degree of Redness

According to the method of Fang Zhaoqin, the forepaw vein images of mice were collected by a stereo microscope at a room temperature of 25°C and 100 lux illumination, and the veins of the second and third palm toe pads of the forepaw were analyzed by the software of Photoshop CS. RGB summed value divided by R value was used as evaluation indicator.

#### Grip Strength Test (GST)

The process was slightly modified as described in the previous literature (Maurissen et al., 2003). The grip strength of each strain of experimental animals was measured by a hand-held HP-100 digital display push/pull meter, and the grip strength of each mouse was recorded, and the measurement was repeated for five times to take the average highest grip strength as the final value. In this way, the value of the grip strength under the natural reaction conditions of the mouse was evaluated.

#### Exhaustive Swimming Test (EST)

The process was slightly modified as described in the previous study (Jin and Wei, 2011; Qi et al., 2014; Lamou et al., 2016). The bear load was applied in accordance with 10–15% of the body weight of the mouse, and the paper clip was fixed at 1–3 cm in the tail of the mouse. The animals were placed in clear glass beaker containing about 3 L of water at a water depth of about 15 cm and the water temperature was maintained at 25 ± 1°C. The mice were observed and recorded from being put in the water to being exhausted.

#### New Object Recognition Test (NOR)

This experiment is based on the rodent’s preference for novel objects, and improved with reference to previous literature. The experiment is divided into three stages. The first stage is the adaptation stage: put the mice in three boxes without any objects and allow them to explore freely for 10 min. The mice were then returned to their original cages. The second stage was the memory stage: objects of the same shape and color were placed symmetrically on either side of the three boxes. The mice were put into the box and allowed to explore freely for 10 min. When the exploration was over, the mice were returned to the original cage. The third stage is the testing stage. After 6 h, one of the objects is replaced with a new object with a different color and shape but a similar size. The number of times the mice explored the new and old objects within 5 min is recorded. The recognition index is used to determine the recognition and memory ability of mice. New object recognition index: the number of times to explore new objects/total number of times to explore new and old objects.

### Fecal Mouse Microbiota Sampling

The process was slightly modified as described in the previous literature (Zheng et al., 2019; Zhu et al., 2020). Fresh fecal pellets were collected sterilely from Balb/C mice at the same time of the day and then placed immediately on dry ice before transfer into −80°C. Mice were mixed across experimental groups before the start of antibiotic treatments to mitigate cage bias. Pellets were also collected every other week post-treatment for 16S rDNA sequencing as detailed below. DNA extraction kit (E.Z.N.A. ^®^ soil DNA kit) was used to extract DNA from corresponding samples. The concentration and purity were determined by the Nano Drop One (Thermo Fisher Scientific, MA, United States).

### 16S rDNA High-Throughput Sequencing

DNA isolation from microbiota samples was performed as above. The length and concentration of the PCR product were detected by 1% agarose gel electrophoresis. There is a bright main band between the samples, which can be used for further experiments. A thermos cycler polymerase chain reaction (PCR) System was employed for the amplification of the V3-V4 hypervariable parts of the bacterial 16S rRNA gene *via* primers 338F (5′- ACT​CCT​ACG​GGA​GGC​AGC​AG-3′) and 806R (5′-G GGACTACHVGGGTWTCTAAT-3′). PCR products was mixed in equal density ratios according to the Gene Tools Analysis Software. The PCR reaction procedure: denaturation at 95°C for 3 min, 27 cycles of 30 s at 95°C, annealing at 55°C for 30 s, and extension at 72°C for 45 s, and a final extension at 72°C for 10 min. Then, mixture PCR products was purified with EZNA Gel Extraction Kit (Omega, United States). Appropriate primers were selected for each project. Purified amplicons were pooled in equimolar and paired-end sequenced (2 × 300) on an Illumina MiSeq platform (Illumina, San Diego, United States) according to the standard protocols by Majorbio Bio-Pharm Technology Co. Ltd (Shanghai, China).

Raw fastq files were quality-filtered through Trimmomatic and merged through FLASH according to the following criteria: 1) Within the 50 bp sliding window, the reads of any part with an average quality score <20 will be truncated. 2) Sequences with an overlap length being longer than 10 bp were merged according to their overlap with mismatch and shall not exceed 2 bp. 3) Sequences of each sample were separated according to barcodes (exactly matching) and Primers (allowing two nucleotide mismatching), and reads containing ambiguous bases were removed. Operational taxonomic units (OTUs) were clustered with 97% similarity cutoff using UPARSE (version 7.1 http://drive5.com/uparse/) with a novel “greedy” algorithm that performs chimera filtering and OTU clustering simultaneously. The taxonomy of each 16S rRNA gene sequence was analyzed by RDP Classifier algorithm (http://rdp.cme.msu.edu/) against the Silva (SSU123) 16S rRNA database using confidence threshold of 70%.

### HPLC Analysis

The fingerprint of JPJY were analyzed by using HPLC analysis (shown in Supplementary Figure S1). The HPLC analysis was performed on a Waters 2,695 Alliance HPLC system (Waters Corp., Milford, MA, United States), equipped with a quaternary pump solvent management system. Chromatographic separations were performed on Apollo C18 column (5 μm, 250 mm × 4.6 mm) and RP C18 guard column (20 mm × 3.9 mm, 5 μm). Flow rate and column temperature were set at 1 ml min^−1^ and 25°C, respectively.

The DAD detector was set at 220 nm for acquiring chromatograms. The mobile phase was composed of D (acetonitrile) and B (water) with a gradient elution: 0–35 min, 5–35% D; 35–43 min, 35–95% D; 43–48 min, 95% D. HPLC-DAD chromatographic data of the 10 tested samples were submitted for analysis by using the professional software “Similarity Evaluation System for Chromatographic Fingerprint of TCM” (Version 2004 A) to extract the mean chromatogram and the similarities. Mouse serum washomogenized at low temperature and centrifuged to prepare a homogenate. 2,4-dinitrofluorobenzene (DNFB) was used as derivatization Reagents. Chromatographic separations were performed on Diamonsil C18 column.

Diamino acids are completely separated within 18 min. Within the range of 0.50–100 ug/ml, the linear relationship is good (*R* ^2^ = 0.99).

### Statistics Analyses

SPSS 19.0 was used for statistical analysis. Multiple comparisons were performed by one-way ANOVA, followed by the Tukey’s multiple comparisons test. All data are presented as Mean ± SEM and were statistically significant at the 5% level unless otherwise stated.

## Results

### Effect of Different Dose of JPJY Administration on Immobility Time in TST

**FIGURE 1:**
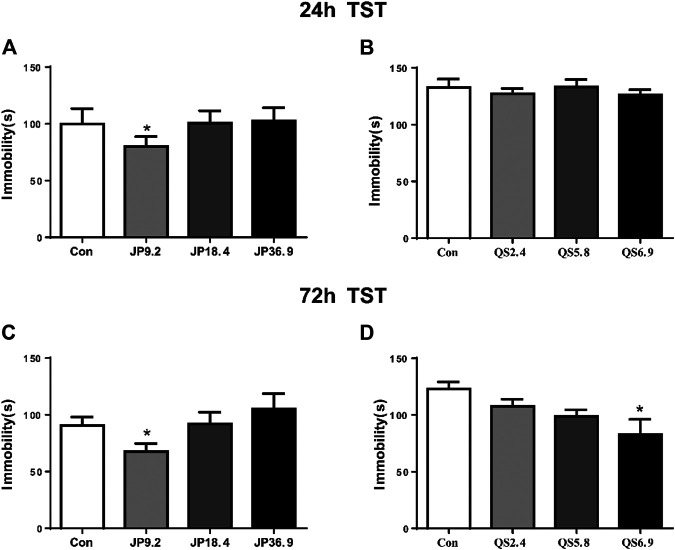
Screen of effective antidepressant dose of JPJY and QS. Control animals (Con) received vehicle treatment, the doses of JPJY with 9.2, 18.4 and QS with 2.4, 5.8, and 6.9 g/kg were used for test. **(A)** Tail suspension test was carried out at 24 h after JPJY administration. Immobility time was measured for the last 4 min during the 6 min testing time [ANOVA, F (3, 37) = 3.379, *p* < 0.05, and *n* = 9–12). **(B)** Tail suspension test at 24 h after QS administration [ANOVA, F (3, 28) = 0.3887, *p* > 0.05, and *n* = 8]. **(C)** Tail suspension test at 72 h after JPJY administration [ANOVA, F (3, 39) = 3.140, *p* < 0.05, and *n* = 8–10]. **(D)** Tail suspension test at 72 h after QS administration [ANOVA, F (3, 28) = 3.975, *p* < 0.05, and *n* = 8]. Data represent mean ± SEM, *p < 0.05.

To examine whether different dose of JPJY and QS attenuated depression in mice under stress conditions, mice were orally administered JPJY and QS for 2 weeks, and TST was performed. Compared with stress mice, 9.2 g/kg of JPJY treatment could significantly reduce immobility time in the TST (*p* < 0.05) at 24 and 72 h (*p* < 0.05) (Figures 1A,C). Meanwhile the dose of 6.9 g/kg QS treatment could effectively decrease the immobility time in the TST at 72 h (*p* < 0.05) but not at 24 h (Figures 1B,D). Therefore, 9.2 g/kg of JPJY and 6.9 g/kg QS was an optimal dose that effectively elicit antidepressant response. JPJY and QS treatment did not affect the time spent in central area or total distance in the open field test (data not shown). Thus, the dose of 9.2 g/kg of JPJY and 6.9 g/kg QS were an effective dose and would be used in the following experiments.

### Effect of JPJY Administration on Fatigue-Like, Depressed Phenotype and Cognitive Function

**FIGURE 2:**
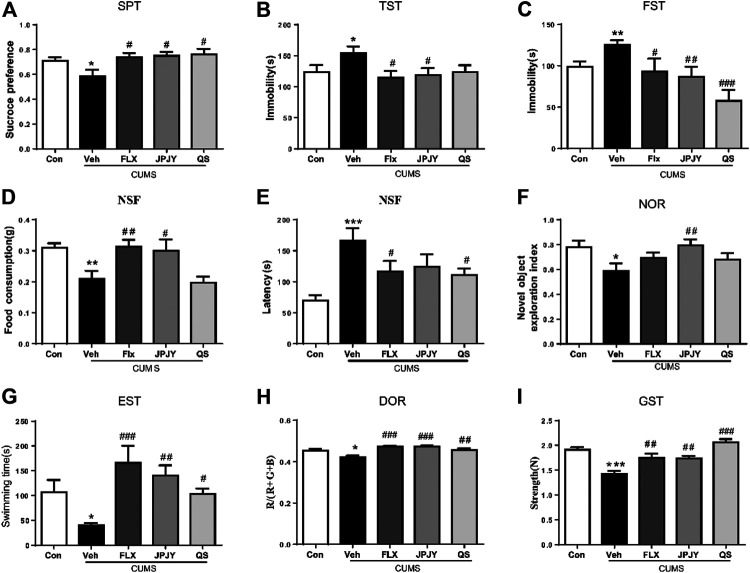
The anti-fatigue and antidepressant behaviors following drug administration in mice exposed to chronic mild stress. Mice were exposed to chronic unpredictable mild stress (CUMS), and received treatment of Veh (Vehicle), FLX, JPJY and QS. Control (Con) mice were not exposed to stress but received vehicle treatment. Behaviors were tested in 2 days after drug administration. **(A)** Group difference in sucrose preference test [F (4, 57) = 3.268, *p* < 0.05 and *n* = 11–15]. **(B,C)** Group difference in immobility time in TST [F (4, 41) = 2.875, *p* < 0.05 and *n* = 8–12] or FST [F (4, 47) = 5.849, *p* < 0.05 and *n* = 9–12]. **(D,E)** Group difference in latency [F (4, 54) = 6.003, *p* < 0.05 and *n* = 9–15] and food consumption [F (4, 50) = 6.114, *p* < 0.05 and *n* = 9–15] in NSF test. **(F)** Group difference in new object recognition index in NOR [F (4, 52) = 2.633, *p* < 0.05 and *n* = 11–12]. **(G)** Group difference in latency in exhausted swim test [F (4, 44) = 5.009, *p* < 0.05 and *n* = 9–11]. **(H)** Group difference in RGB summed value divided by R value in degree of redness [F (4, 40) = 8.946, *p* < 0.05 and *n* = 8–10]. **(I)** Group difference in strength in grip strength [F (4, 62) = 15.96, *p* < 0.05 and *n* = 11–15]. One-way ANOVA, *p < 0.05, **p < 0.01, ***p < 0.001 vs. CTL; ^#^ *p* < 0.05, ^##^ *p* < 0.01, ^###^ *p* < 0.001 vs. Veh. Data represent means ± SEM.

Animals displayed depressive responses, indicated by increase in sucrose preference, increase in immobility time in TST, FST, reduce in food consumption and increase in latency of NSF (all *p* < 0.05, vs. CTL). Compared to vehicle group, JPJY and FLX treatment could significantly reduce sucrose preference (*p* < 0.05 vs. Veh), decrease the immobility time in TST (*p* < 0.05 vs. Veh) and FST (*p* < 0.05 vs. Veh). QS treatment could not alter the immobility in the TST (*p* > 0.05 vs. Veh) (Figures 2A–C). Although animals still displayed depressive-like behavior in the NSF, FLX and JPJY treatment restored their food consumption (*p* < 0.05 vs. Veh) and decreased latency (*p* < 0.05 vs. Veh) (Figures 2D,E). Mice received CUMS for 4 weeks, novel object exploration index of NOR were decreased (*p* < 0.05, vs. CTL). Among these treatment groups, JPJY treatment could effectively ameliorate cognitive function in CUMS mice, and increase novel object exploration index of NOR (*p* < 0.05 vs. Veh, Figure 2F). Additionally, mice also displayed fatigue-like responses, indicated by decrease latency of exhausted swim test, reduced RGB/R in degree of redness and decrease strength in grip strength (all *p* < 0.05, vs. CTL). Compared to the vehicle group, FLX, JPJY, and QS treatment reversed the fatigue-like behaviors. In summary, repeated administration of JPJY displayed full spectrum of antidepressant effect, whereas fluoxetine and QS failed to improve the cognitive function.

### Effect of JPJY Administration on Gut Microbiota Diversity

**FIGURE 3:**
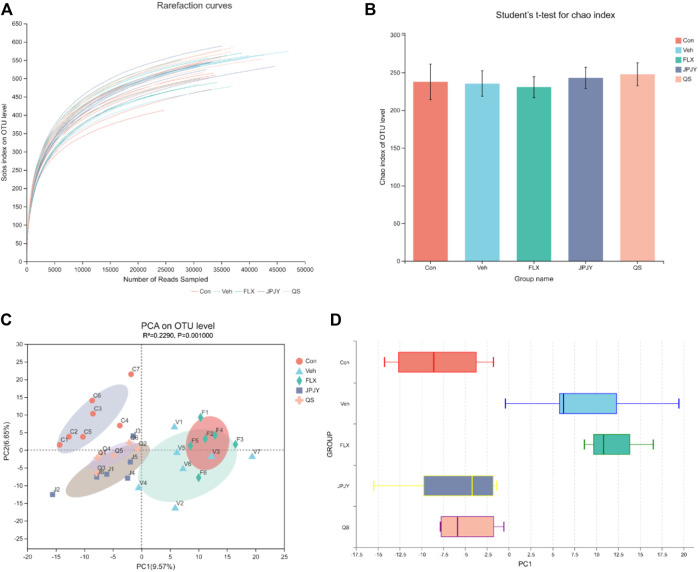
Analysis of gut microbiota following drug treatment. Microbiota composition in feces of mice treated with drugs were analyzed using next generation sequencing. **(A)** Sobs rarefaction curve. **(B)** Alpha diversity index (Chao estimator) analysis for each group. **(C)** PCA (Principal Component Analysis) on OUT level. **(D)**Plots shown were generated using the PCA. The boxplot in the figure represents the discrete distribution of different groups of samples on the PC1 axis.

By pyrosequencing bacterial 16S rDNA (v4-v5 region) in cecal feces, we detected the effect of drugs on gut microbiota composition. In order to analyze the effect of drug treatment on the composition of gut microbiota, we sequenced 1,665,887 clean reads from 32 samples. A flat curve indicates that the sequencing data volume is sufficient (Figure 3A). The Chao index of alpha Diversity did not change significantly (Figure 3B, *p* > 0.05). The results of beta diversity analysis showed that the microbiota of control was significantly different in that of vehicle and fluoxetine, but not in JPJY and QS. It was found that the microbiota population of the JPJY and QS treatment group were significantly separated from that of vehicle group (Figures 3C,D), and FLX treatment group was close to vehicle group, indicating that the composition of the cecal fecal microbiota was changed after JPJY and QS treatment, but not FLX treatment.

### Effect of JPJY Administration on Phylum and Genus Level of Gut Microbiota

**FIGURE 4:**
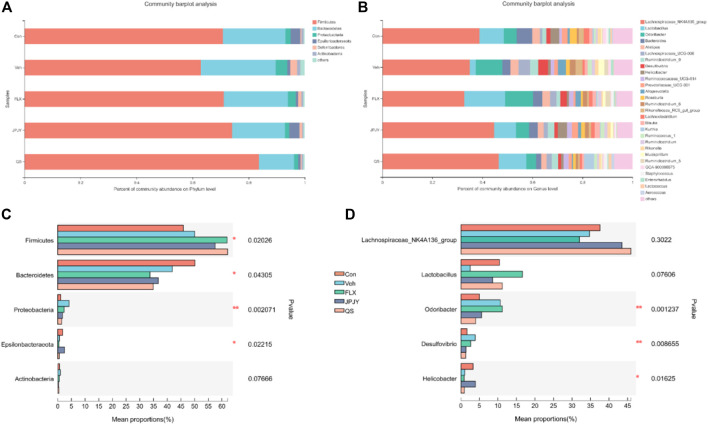
Related phylum and genus level bacterial abundance. **(A)** Bacterial taxonomic profiling in the phylum level of gut microbiota from different groups. **(B)** Bacterial taxonomic profiling in the genus level of intestinal bacteria from different groups. **(C)** Kruskal-Wallis H test bar plot of *Firmicutes*, *Proteobacteria*, *Bacteroidetes*, *Actinobacteria* and *Deferribacteres* in phylum level. **(D)** Kruskal-Wallis H test bar plot of *Lactobacillus,* Lachnospiraceae*_NK4A136_group*, *Odoribacter*, *desulfovibrio*, and *helicobacter* in genu level.

Taxonomic shifts were also investigated, and at the phylum level the cecal fecal microbiota was dominated by *Firmicutes*, *Bacteroidetes*, *Proteobacteria*, *Deferribacteres*, *Epsilonbacteraeota* and *Actinobacteria*, showing significant changes in these microbiota among the mice groups (Figure 4A). Compared to the control group, the vehicle group exhibited a markedly increased abundance of *Firmicutes*, *Proteobacteria*, and *Actinobacteria*, and reduced abundance of *Epsilonbacteraeota* and *Bacteroidetes* (Figure 4C). Alterations were detected on the levels of order, class, family, and genus (Figure 4B). At the genus level, compared with control group, the abundance of *Odoribacter* and *Desulfovibrio* of the Veh control group increased, and the abundance of *Lactobacillus* and *helicobacter* decreased. The JPJY treatment could reverse the changes of these bacteria, and make its abundance similar to the control group. QS treatment could not reverse the abundance of *helicobacter*, and fluoxetine treatment could not reverse the *Odoribacter* and *helicobacter* (Figures 4B,D). The above result suggests that antidepressant effects of JPJY are likely due to a difference in the relative abundance of *Lactobacillus* and *Desulfovibrio.*

### The Core Gut Microbiota of Depression Modulated by JPJY

**FIGURE 5:**
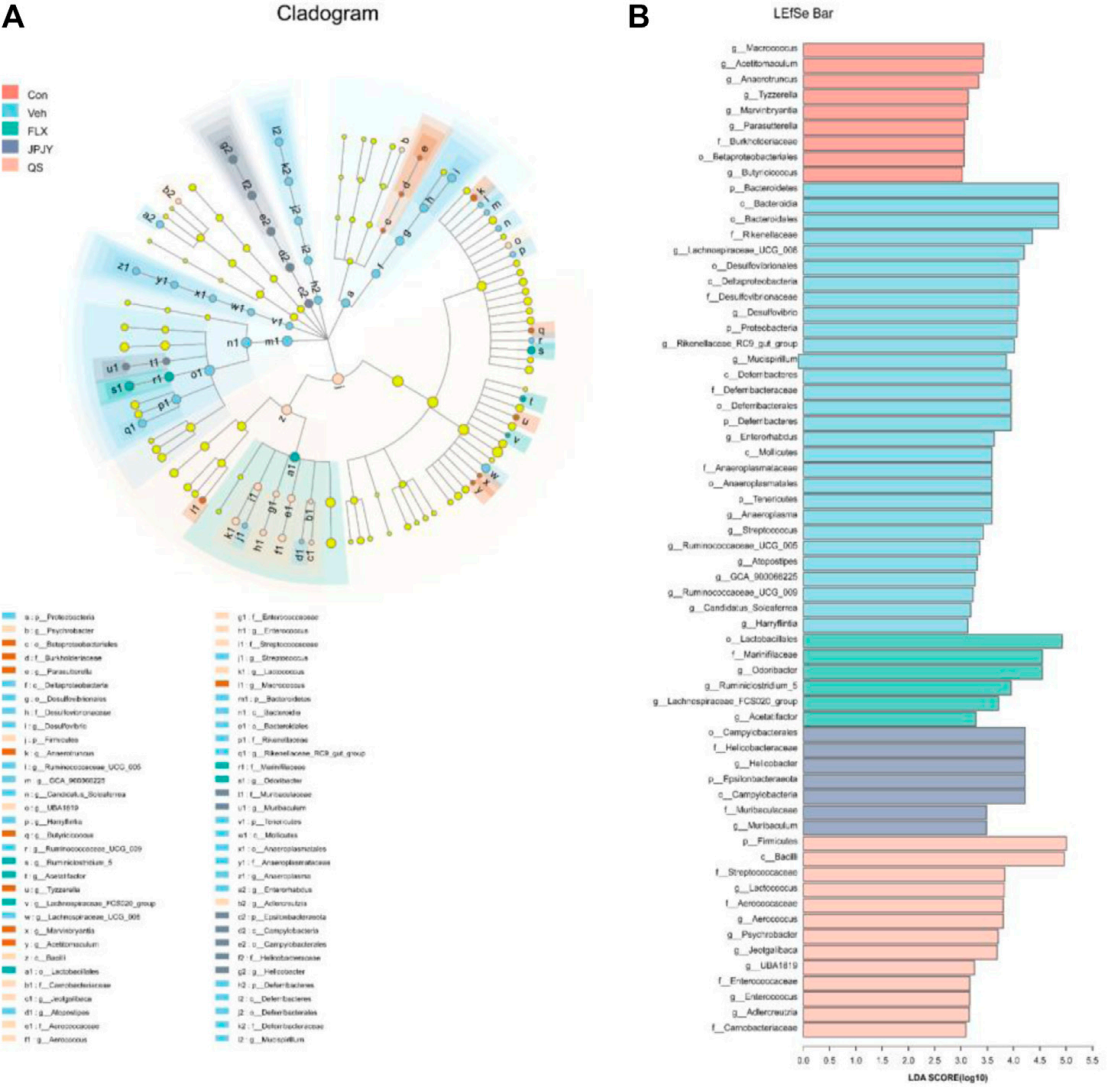
Analysis of gut microbiota following drug treatment. **(A)** Cladogram for taxonomy generated through LEfSe analysis showing prominent shifts in the gut microbiota in each group (score >3). **(B)** Linear discriminant analysis (LDA) scores (log10) of taxa (score >3).

We further used LEfSe analysis to highlight the bacterial phenotypes that cause the variation of gutmicrobiota and listed 62 gut bacterial clades with major differences (Figures 5A,B). In the CTL group, the *Macrococcus* and *Acetitomaculum* were the most abundant. In addition, the LDA score of the *Bacteroidetes* and *Proteobacteria* were enriched by vehicle. In the fluoxetine group, the LDA score of the *Lactobacillales* and *Odoribacter* were found to be enriched. In the JPJY-treated group, *Helicobacter* and *Epsilonbacteraeota* be the abundant one. *Firmicutes* and *Bacilli* were enriched in QS group.

### Effects of JPJY Administration on Concentration of GABA in Mice Serum

**FIGURE 6:**
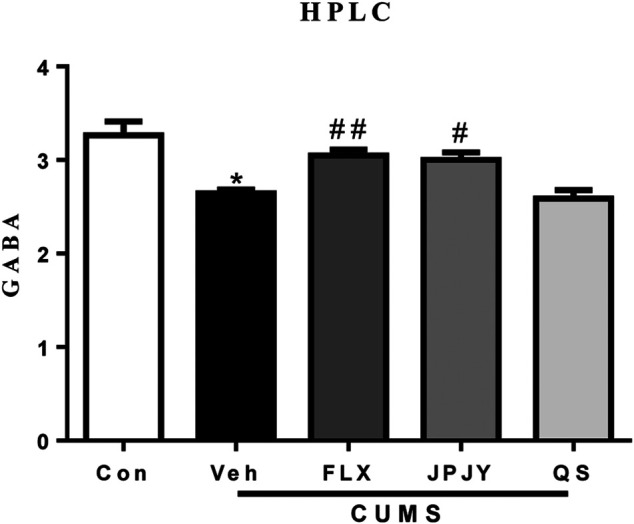
The expressions of GABA in serum were reversed in mice exposed to chronic mild stress. Mice were exposed to chronic unpredictable mild stress (CUMS), and received treatment of Veh (Vehicle), FLX, JPJY and QS. Control (Con) mice were not exposed to stress but received vehicle treatment [F (4, 15) = 9.628, *p* < 0.001].

The results showed that the concentration of GABA significantly reduced in mice under chronic stress conditions (Figure 6; *p* < 0.05 vs. CTL). After chronic treatment of JPJY, the concentration of GABA was reversed (Figure 6; *p* < 0.05 vs. Veh), which was similar to fluoxetine treatment (Figure 6; *p* < 0.05 vs. Veh). However, the concentration of GABA in QS-treated group was no alteration (Figure 6; *p* > 0.05 vs. Veh).

### Effects of JPJY Administration on GluR1 and p-Tau Expression

**FIGURE 7:**
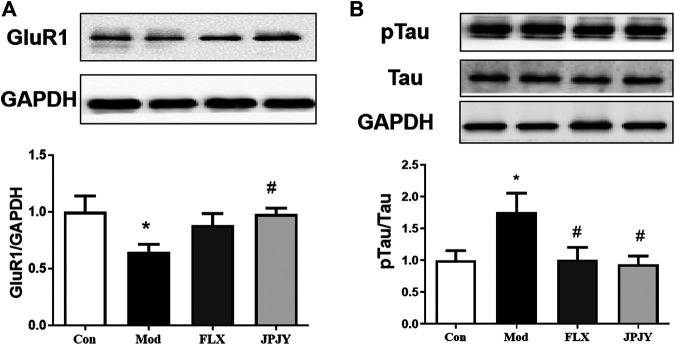
The expressions of GluR1 and p-Tau/Tau in hippocampus were reversed in mice exposed to chronic mild stress. Mice were exposed to chronic unpredictable mild stress (CUMS), and received treatment of Veh (Vehicle), FLX, JPJY and QS. Control (Con) mice were not exposed to stress but received vehicle treatment. **(A)** The expression of Glur1 F (3, 13) = 2.569; *p* = 0.00993. **(B)** The expression of p-Tau/Tau, F (3, 11) = 1.807; *p* < 0.05, *n* = 4–5/group.

The expressions of GluR1 and p-Tau in CUMS mice hippocampus was determined. The results showed that the expression of GluR1 significantly reduced (Figure 7A; *p* < 0.05 vs. CTL), and p-Tau significantly increased by chronic stresses (Figure 7B; *p* < 0.05 vs. CTL). After chronic treatment of JPJY, the expression of GluR1 and p-Tau were both significantly reversed (Figures 7A,B; *p* < 0.05 vs. Veh), but fluoxetine-treated group showed no significant change (Figures 7A,B; *p* < 0.05 vs. Veh).

## Discussion

Our present study firstly demonstrated that JPJY decoction as well as its domain component QS could produce antidepressant-like effect both in normal and stress induced Balb/c mice. To investigate its potential mechanism, gut microbiome was measured and found JPJY treatment and normal mice reveal similar distribution of microbiome. JPJY modulated gut microbiota diversity and richness, it significantly reduced the relative abundance of *Proteobacteria*, and increase the abundance of *Lactobacillus*. Besides, we also found that GABA concentration was significantly reduced after chronic stress while JPJY and Flx (except QS) could significantly increase the GABA concentration in serum of mice. Meanwhile, chronic stress caused decreased GluR1 and p-Tau expression in hippocampus and increased GluR1 and p-Tau expression after JPJY and FLX treatment as compared with the control groups. Tau was referred to play an important role in memory and we found that JPJY reversed the phosphorylation expression of Tau in hippocampus. To sum up, JPJY could produce antidepressant-like effect by regulating gut microbiota to modulate hippocampus GABA pathway.

Previous study found that Balb/c mice showed fatigue-like behaviors after under chronic unpredictable mild stress. This was also demonstrated in our experiments. JPJY, QS and FLX all reversed fatigue-like behaviors. The aqueous extract of *Astragali Radix* could enhance the anti-fatigue effect of mice through anti-free radical action. *Astragali Radix* reduces fatigue and improves sleep quality of adults with long-term fatigue (Sun et al., 2016; Baek et al., 2018; Lee et al., 2019). Song Li Tao et al. found that *Radix pseudostellariae* could prolong the duration of load-bearing swimming in mice and the survival time of mice under hypoxia state, proving that Taizishen had anti-fatigue and anti-stress functions. However, only JPJY ameliorated cognitive function in mice, and increased novel object exploration index of NOR. It may be related to Yuan Zhi. There are results suggesting that Senegenin in Yuan Zhi not only had the characteristics of cell protection, and neurogenic properties, but also increased the expression of cardinal growth proteins, which is essential for neural plasticity (Jesky and Chen, 2016). Tenuigenin protects dopaminergic neurons from inflammation by inhibiting the activation of NLRP3 inflammasome in microglia (Fan et al., 2017). Researchers have found that intragastric administration of Senegenin attenuated HIR-induced cognitive impairment in a dose and time dependent manner in rat hippocampus (Xie et al., 2012). Different components showed different functional effects, which resulted in therapeutic effect of depression, cognition deficit and fatigue (considered as qi deficiency in Chinese Medicine).

A large amount of researches have confirmed that depression was related to changes in the composition of the gut microbiota, usually in the form of changes in richness and diversity (Winter et al., 2018; Reid, 2019). Taxonomic shifts were investigated, the cecal gut microbiota was dominated by *Firmicutes, Bacteroidetes* and *Proteobacteria* at the phylum level, showing great changes among the mice groups. We found that the relative abundance of *Firmicutes*, *Proteobacteria* and *Actinobacteria* in the Veh group increased, while the relative abundance of *Bacteroidetes* decreased. Studies have found that depressive-like rats had the relative abundances of *Bacteroidetes* significantly increased, while the relative abundance that of *Firmicutes* reduced significantly (Yu et al., 2017; Lach et al., 2018). Jiang et al. found that compared with healthy control individuals, there was an increase in *Bacteroidetes*, *Proteobacteria* and *Actinobacteria* in subjects with major mental disorder (Jiang et al., 2015). In the study of Zheng et al., it was found that compared with healthy controls, the relative abundance of *Bacteroidetes* in MDD subjects was reduced, and the relative abundance of *Actinobacteria* was increased in MDD subjects. There is no significant difference in the overall relative abundance of *Firmicutes* between the two groups (Zheng et al., 2016). This difference may be due to differences in sample size, demographic and clinical characteristics of recruited MDD subjects and/or statistical methods used to identify gut microbiota related to MDD, or to differences in the composition of humans and mice gut microbiota. There is evidence that the *Proteobacteria* can reflect microbial dysbiosis or unstable intestinal microbial community structure. Healthy mammalian gut also contains symbiotic bacteria of *Proteobacteria*. When the number of these bacteria is small, these bacteria benign, but under certain gut environment, they can become gut microbes that can cause inflammation (Shin et al., 2015). In this experiment, After continuous administration of drugs, the relative abundance of *Proteobacteria* significantly reduced. Consequently, these findings suggest that depression is linked to changes in the composition of gut microbiota.

At the genus level, mild stress treatment enhanced the abundance of *Odoribacter* and decreased the abundance of *Lactobacillus* and *Lachnospiraceae_NK4A136* group, which had high abundance in the JPJY group. Tiansi Liquid has an antidepressant-like effect, it also significantly increased the relative abundance of *Lachnospiraceae_NK4A136* group in the feces (Cheng et al., 2018). C57BL/6J mice were treated with azoxymethane and dextran sulfate sodium was lower in *Lactobacillus* than the control group. The probiotic *S. boulardii* has been shown to inhibit pro-inflammatory cytokines. After *S. boulardii* exposure, the percentage contributions of *Lactobacillus* and *Lachnospiraceae_NK4A136_group* increased (Wang et al., 2019). *Odoribacter* is a normal member of the human gut microbiota, which increased significantly in the stressed mice (Hufeldt et al., 2010). These suggesting that *Odoribacter, Lachnospiraceae_NK4A136* group and *Lactobacillus* may be opportunistic pathogens in the course of depression.

Researchers found that the relative abundance levels of *Bacteroides* are negatively correlated with GABA (Strandwitz et al., 2019; Duranti et al., 2020). But in our experiment *Bacteroides* did not change negatively correlated with depression behaviors. The treatment of JPJY, QS and FLX increased the relative abundance of *Lactobacillus* after the CUMS. Many data show that *Lactobacillus* improve depression-like behavior in mice through neurotransmitter, HPA axis, immunity and other ways. It is worth noting that it has been reported that treatment of mice with the *Lactobacillus rhamnosus* (*JB-1*) reduces stress and depression-like behavior in a vagal dependent way (Bravo et al., 2011). It was accompanied by changes of GABA receptors expression in many brain regions, including the amygdala and hippocampus. Thus, *Lactobacillus* beneficial to the inclusion/production of GABA can be regarded as a transmitter of neuroactive compounds. It was speculated that *lactobacillus* produced the neurotransmitter GABA and played a role in the antidepressant effects of JPJY.

Neurotransmitters, including GABAergic receptors, are involved in the pathophysiology of depression (DM et al., 2020). Bursting of glutamatergic signals in mPFC, causing steady-state synaptic plasticity, resulting in rapid antidepressant effects (Hare et al., 2019). In addition, the excitatory GABAergic neurons in mPFC are also required for the rapid antidepressant effect (DM et al., 2020). Moreover, NMDAR antagonists can initially modulate the spontaneous discharge of glutamatergic and GABAergic receptors (Constantinidis and Goldman-Rakic, 2002). In this study, JPJY reversed the down-regulation of GABA in serum and expression of GluR1 in hippocampus, which were reduced by stressors. The data showed bursting of GABAergic and Glur1 receptor plays role in antidepressant-like effects of JPJY.

Tau protein is most abundantly expressed in the axons of central nervous system neurons (Jameson et al., 1980). More specifically, in the “pre-tangled” stage of neurofibrillary degeneration, abnormal phosphorylation, aggregation and proteolysis of tau protein have been proved by neuropathology to be the early and key events of AD (Simic, 2002). The latest data obtained indicate that after injecting tau oligomers or aggregates into wild-type or mutant MAPT transgenic mice, tau pathology can indeed be induced and spread (Iba et al., 2013), and tau aggregates can be transferred from a cell *in vitro* To another cell (Frost et al., 2009) and in the body (Liu et al., 2017). In addition, antibodies that block tau accumulation seeds have been shown to improve cognition *in vivo* (Yanamandra et al., 2013). In this study, JPJY reduced the phosphorylation expressions of Tau in hippocampus, which was increased by stressors. Therefore, inhibiting expression of Tau in hippocampus participated in critical role in improving of cognition.

In summary, the present study demonstrated that the JPJY has significant antidepressant-like efficacy and effectively alleviated behavioral deficits in CUMS animals, and it was also indicated that JPJY modulate the gut–brain axis response through gamma aminobutyric acid (GABA). Repeated administration of JPJY displayed a good improvement in cognitive function, while fluoxetine and QS did not. It was speculated that *lactobacillus* produced the neurotransmitter GABA to exert antidepressant effects. However, our experiment also had some limitations. We did not directly link GABA with gut microbiota, nor did we analyze the how NR1 and Tau affect GABA expression in hippocampus after JPJY treatment.

## Data Availability

The datasets presented in this study can be found in online repositories. The names of the repository/repositories and accession number(s) can be found below: Sequence Read Archive SUB9065437.

## References

[B91] Barrett E. Ross R. P. O'Toole P. W. Fitzgerald G. F. Stanton C. (2012). gamma-Aminobutyric acid production by culturable bacteria from the human intestine. J. Appl. Microbiol. 113 (2), 411–417. 10.1111/j.1365-2672.2012.05344.x 22612585

[B1] Baek Y. Kim H. Mun S. Lee S. (2018). Three-component herbal tea alleviates prolonged fatigue and improves sleep quality: a randomized controlled pilot study. Explore 14 (6), 420–423. 10.1016/j.explore.2018.05.001 30482676

[B2] Bravo J. A. Forsythe P. Chew M. V. Escaravage E. Savignac H. M. Dinan T. G. (2011). Ingestion of Lactobacillus strain regulates emotional behavior and central GABA receptor expression in a mouse *via* the vagus nerve. Proc. Natl. Acad. Sci. 108 (38), 16050–16055. 10.1073/pnas.1102999108 21876150 PMC3179073

[B3] Browne H. P. Forster S. C. Anonye B. O. Kumar N. Neville B. A. Stares M. D. (2016). Culturing of ‘unculturable’ human microbiota reveals novel taxa and extensive sporulation. Nature 533 (7604), 543–546. 10.1038/nature17645 27144353 PMC4890681

[B4] Chen G. Guo X. (2017). Neurobiology of Chinese herbal medicine on major depressive disorder. Int. Rev. Neurobiol. 135, 77–95. 10.1016/bs.irn.2017.02.005 28807166

[B5] Cheng D. Chang H. Ma S. Guo J. She G. Zhang F. (2018). Tiansi liquid modulates gut microbiota composition and Tryptophan(-)Kynurenine metabolism in rats with hydrocortisone-induced depression. Molecules. 23 (11), 2832. 10.3390/molecules23112832 PMC6278342 30384480

[B76] Constantinidis C. Goldman-Rakic P. S. (2002). Correlated discharges among putative pyramidal neurons and interneurons in the primate prefrontal cortex. J. Neurophysiol. 88 (6), 3487–3497. 10.1152/jn.00188.200288 12466463

[B6] Cryan J. F. Dinan T. G. (2012). Mind-altering microorganisms: the impact of the gut microbiota on brain and behaviour. Nat. Rev. Neurosci. 13 (10), 701–712. 10.1038/nrn3346 22968153

[B7] Dhakal R. Bajpai V. K. Baek K. H. (2012). Production of gaba (γ - Aminobutyric acid) by microorganisms: a review. Braz. J. Microbiol. 43 (4), 1230–1241. 10.1590/s1517-83822012000400001 24031948 PMC3769009

[B8] Duranti S. Ruiz L. Lugli G. A. Tames H. Milani C. Mancabelli L. (2020). Bifidobacterium adolescentis as a key member of the human gut microbiota in the production of GABA. Sci. Rep. 10, 14112. 10.1038/s41598-020-70986-z 32839473 PMC7445748

[B9] Fan Z. Liang Z. Yang H. Pan Y. Zheng Y. Wang X. (2017). Tenuigenin protects dopaminergic neurons from inflammation *via* suppressing NLRP3 inflammasome activation in microglia. J. Neuroinflammation 14 (1), 256. 10.1186/s12974-017-1036-x 29262843 PMC5738892

[B10] Felger J. C. Cole S. W. Pace T. W. W. Hu F. Woolwine B. J. Doho G. H. (2012). Molecular signatures of peripheral blood mononuclear cells during chronic interferon-α treatment: relationship with depression and fatigue. Psychol. Med. 42 (8), 1591–1603. 10.1017/s0033291711002868 22152193 PMC3433045

[B111] Frost B. Jacks R. L. Diamond M. I. (2009). Propagation of tau misfolding from the outside to the inside of a cell. J. Biol. Chem. 12845–12852. 10.1074/jbc.M808759200284 19282288 PMC2676015

[B11] Fujisaka S. Avila-Pacheco J. Soto M. Kostic A. Dreyfuss J. M. Pan H. (2018). Diet, genetics, and the gut microbiome drive dynamic changes in plasma metabolites. Cel Rep. 22 (11), 3072–3086. 10.1016/j.celrep.2018.02.060 PMC5880543 29539432

[B12] Fung T. C. Olson C. A. Hsiao E. Y. (2017). Interactions between the microbiota, immune and nervous systems in health and disease. Nat. Neurosci. 20 (2), 145–155. 10.1038/nn.4476 28092661 PMC6960010

[B92] Gerhard D. M. Pothula S. Liu R.J. Wu M. Li X. Y. Girgenti M. J. (2020). GABA interneurons are the cellular trigger for ketamine's rapid antidepressant actions. J. Clin. Investig. 130 (3), 1336–1349. 10.1172/JCI130808130 31743111 PMC7269589

[B95] Hare B. D. Shinohara R. Liu R. J. Pothula S. DiLeone R. J. Duman R. S. (2019). Optogenetic stimulation of medial prefrontal cortex Drd1 neurons produces rapid and long-lasting antidepressant effects. Nat. Commun. 10, 223. 10.1038/s41467-018-08168-9 30644390 PMC6333924

[B13] Hufeldt M. R. Nielsen D. S. Vogensen F. K. Midtvedt T. Hansen A. K. (2010). Variation in the gut microbiota of laboratory mice is related to both genetic and environmental factors. Comp. Med. 60 (5), 336–347. 10.1152/ajpgi.00026.2020 21262117 PMC2958200

[B97] Iba M. Guo J. L. McBride J. D. Zhang B. Trojanowski J. Q. Lee V. M. (2013). Synthetic tau fibrils mediate transmission of neurofibrillary tangles in a transgenic mouse model of Alzheimer's-like tauopathy. J. Neurosci. 1024–1037. 10.1523/JNEUROSCI.2642-12.201333 23325240 PMC3575082

[B87] Jameson L. Frey T. Dalldorf F. Caplow M. (1980). Inhibition of microtubule assembly by phosphorylation of microtubule-associated proteins. Biochem. 2472–2479. 10.1021/bi00552a02719 7387985

[B14] Jesky R. Chen H. (2016). The neuritogenic and neuroprotective potential of senegenin against Abeta-induced neurotoxicity in PC 12 cells. BMC Complement. Altern. Med. 16, 26. 10.1186/s12906-016-1006-3 26803813 PMC4724108

[B15] Jiang H. Ling Z. Zhang Y. Mao H. Ma Z. Yin Y. (2015). Altered fecal microbiota composition in patients with major depressive disorder. Brain Behav. Immun. 48, 186–194. 10.1016/j.bbi.2015.03.016 25882912

[B16] Jin H. M. Wei P. (2011). Anti-fatigue properties of tartary buckwheat extracts in mice. Int. J. Mol. Sci. 12 (8), 4770–4780. 10.3390/ijms12084770 21954324 PMC3179131

[B17] Kali A. (2016). Psychobiotics: an emerging probiotic in psychiatric practice. Biomed. J. 39 (3), 223–224. 10.1016/j.bj.2015.11.004 27621125 PMC6140288

[B18] Kelly J. R. Borre Y. O' Brien C. Patterson E. El Aidy S. Deane J. (2016). Transferring the blues: depression-associated gut microbiota induces neurobehavioural changes in the rat. J. Psychiatr. Res. 82, 109–118. 10.1016/j.jpsychires.2016.07.019 27491067

[B19] Koren O. Goodrich J. K. Cullender T. C. Spor A. Laitinen K. Kling Bäckhed H. (2012). Host remodeling of the gut microbiome and metabolic changes during pregnancy. Cell 150 (3), 470–480. 10.1016/j.cell.2012.07.008 22863002 PMC3505857

[B120] Kuo Y. H. Tsai W. J. Chiou W. F. Wu T.-S. Chiou W.‐F. (2009). Astragalus membranaceus flavonoids (AMF) ameliorate chronic fatigue syndrome induced by food intake restriction plus forced swimming. J. Ethnopharmacol, 28–34. 10.1016/j.jep.2008.11.025122 19103273

[B20] Lach G. Schellekens H. Dinan T. G. Cryan J. F. (2018). Anxiety, depression, and the microbiome: a role for gut peptides. Neurotherapeutics 15 (1), 36–59. 10.1007/s13311-017-0585-0 29134359 PMC5794698

[B21] Lam R. W. Michalak E. E. Bond D. J. Tam E. M. Axler A. Yatham L. N. (2012). Which depressive symptoms and medication side effects are perceived by patients as interfering most with occupational functioning? Depress. Res. Treat. 2012, 630206. 10.1155/2012/630206 22611491 PMC3350949

[B22] Lamou B. Taiwe G. S. Hamadou A. Abene Houlray J. Atour M. M. (2016). Antioxidant and antifatigue properties of the aqueous extract of moringa oleifera in rats subjected to forced swimming endurance test. Oxid. Med Cell Longev. 2016, 3517824. 10.1155/2016/3517824 26904162 PMC4745945

[B23] Lee J. S. Kim W. Y. Jeon Y. J. Lee S. B. Lee D. S. Son C. G. (2019). Antidepressant-like activity of myelophil *via* attenuation of microglial-mediated neuroinflammation in mice undergoing unpredictable chronic mild stress. Front. Pharmacol. 10, 683. 10.3389/fphar.2019.00683 31263417 PMC6585390

[B24] Li H. Lin S. Qin T. Li H. Ma Z. Ma S. (2017). Senegenin exerts anti-depression effect in mice induced by chronic un-predictable mild stress via inhibition of NF-κB regulating NLRP3 signal pathway. Int. Immunopharmacol. 53, 24–32. 10.1016/j.intimp.2017.10.001 29031144

[B25] Liu P. Zhao H. Luo Y. (2017). Anti-aging implications of *Astragalus* membranaceus (huangqi): a well-known Chinese tonic. Aging Dis. 8 (6), 868–886. 10.14336/ad.2017.0816 29344421 PMC5758356

[B26] Matsumoto M. Ooga T. Kibe R. Aiba Y. Koga Y. Benno Y. (2017). Colonic absorption of low-molecular-weight metabolites influenced by the intestinal microbiome: a pilot study. PLoS One 12 (1), e0169207. 10.1371/journal.pone.0169207 28121990 PMC5266324

[B27] Maurissen J. P. J. Marable B. R. Andrus A. K. Stebbins K. E. (2003). Factors affecting grip strength testing. Neurotoxicol. Teratol. 25 (5), 543–553. 10.1016/s0892-0362(03)00073-4 12972067

[B28] Qi B. Liu L. Zhang H. Zhou G. X. Wang S. Duan X. Z. (2014). Anti-fatigue effects of proteins isolated from Panax quinquefolium. J. Ethnopharmacol. 153 (2), 430–434. 10.1016/j.jep.2014.02.045 24607495

[B29] Reid G. (2019). Disentangling what we know about microbes and mental health. Front. Endocrinol. (Lausanne) 10, 81. 10.3389/fendo.2019.00081 30828318 PMC6384226

[B30] Sarasa S. B. Mahendran R. Muthusamy G. Thankappan B. Selta D. R. F. Angayarkanni J. (2020). A brief review on the non-protein amino acid, gamma-amino butyric acid (GABA): its production and role in microbes. Curr. Microbiol. 77 (4), 534–544. 10.1007/s00284-019-01839-w 31844936

[B31] Savignac H. M. Tramullas M. Kiely B. Dinan T. G. Cryan J. F. (2015). Bifidobacteria modulate cognitive processes in an anxious mouse strain. Behav. Brain Res. 287, 59–72. 10.1016/j.bbr.2015.02.044 25794930

[B131] Sheng R. Xu X. Dai Y. (2011). Polysaccharide of radix pseudostellariae improves chronic fatigue syndrome induced by poly IC in mice. Evid Based Complement Alternat. Med., 840516. 10.1093/ecam/nep2082011 20008077 PMC3137695

[B32] Shin N. R. Whon T. W. Bae J. W. (2015). Proteobacteria: microbial signature of dysbiosis in gut microbiota. Trends Biotechnol. 33 (9), 496–503. 10.1016/j.tibtech.2015.06.011 26210164

[B132] Simic G. (2002). Pathological tau proteins in argyrophilic grain disease. Lancet Neurol. 276. 10.1016/s1474-4422(02)00130-81 12849422

[B33] Song M. T. Ruan J. Zhang R. Y. Deng J. Ma Z. Q. Ma S. P. (2018). Astragaloside IV ameliorates neuroinflammation-induced depressive-like behaviors in mice *via* the PPARγ/NF-κB/NLRP3 inflammasome axis. Acta Pharmacol. Sin 39 (10), 1559–1570. 10.1038/aps.2017.208 29795356 PMC6289360

[B34] Strandwitz P. Kim K. H. Terekhova D. Liu J. K. Sharma A. Levering J. (2019). GABA-modulating bacteria of the human gut microbiota. Nat. Microbiol. 4 (3), 396–403. 10.1038/s41564-018-0307-3 30531975 PMC6384127

[B35] Sun G. G. Shih J. H. Chiou S. H. Hong C. J. Lu S. W. Pao L. H. (2016). Chinese herbal medicines promote hippocampal neuroproliferation, reduce stress hormone levels, inhibit apoptosis, and improve behavior in chronically stressed mice. J. Ethnopharmacol. 193, 159–168. 10.1016/j.jep.2016.07.025 27416803

[B36] Tang J. Xue W. Xia B. Ren L. Tao W. Chen C. (2015). Involvement of normalized NMDA receptor and mTOR-related signaling in rapid antidepressant effects of yueju and ketamine on chronically stressed mice. Sci. Rep. 5, 13573. 10.1038/srep13573 26315757 PMC4551989

[B37] Wang C. Li W. Wang H. Ma Y. Zhao X. Zhang X. (2019). *Saccharomyces* boulardii alleviates ulcerative colitis carcinogenesis in mice by reducing TNF-alpha and IL-6 levels and functions and by rebalancing intestinal microbiota. BMC Microbiol. 19 (1), 246. 10.1186/s12866-019-1610-8 31694526 PMC6836350

[B38] Winter G. Hart R. A. Charlesworth R. P. G. Sharpley C. F. (2018). Gut microbiome and depression: what we know and what we need to know. Rev. Neurosci. 29 (6), 629–643. 10.1515/revneuro-2017-0072 29397391

[B39] Woelfer M. Kasties V. Kahlfuss S. Walter M. (2019). The role of depressive subtypes within the neuroinflammation hypothesis of major depressive disorder. Neuroscience 403, 93–110. 10.1016/j.neuroscience.2018.03.034 29604382

[B40] Xie W. Yang Y. Gu X. Zheng Y. Sun Y. E. Liang Y. (2012). Senegenin attenuates hepatic ischemia-reperfusion induced cognitive dysfunction by increasing hippocampal NR2B expression in rats. PLoS One 7 (9), e45575. 10.1371/journal.pone.0045575 23029109 PMC3448627

[B141] Yanamandra K. Kfoury N. Jiang H. Mahan T. E. Ma S. Maloney S. E. (2013). Anti-tau antibodies that block tau aggregate seeding in vitro markedly decrease pathology and improve cognition in vivo. Neuron, 402–414. 10.1016/j.neuron.2013.07.04680 24075978 PMC3924573

[B41] Yu L. Sun L. Chen S. (2014). Protective effect of senegenin on splenectomy-induced postoperative cognitive dysfunction in elderly rats. Exp. Ther. Med. 7 (4), 821–826. 10.3892/etm.2014.1501 24660030 PMC3961123

[B42] Yu M. Jia H. Zhou C. Yang Y. Zhao Y. Yang M. (2017). Variations in gut microbiota and fecal metabolic phenotype associated with depression by 16S rRNA gene sequencing and LC/MS-based metabolomics. J. Pharm. Biomed. Anal. 138, 231–239. 10.1016/j.jpba.2017.02.008 28219800

[B43] Zheng P. Li Y. Wu J. Zhang H. Huang Y. Tan X. (2019). Perturbed microbial ecology in myasthenia gravis: evidence from the gut microbiome and fecal metabolome. Adv. Sci. 6 (18), 1901441. 10.1002/advs.201901441 PMC6755540 31559142

[B44] Zheng P. Zeng B. Zhou C. Liu M. Fang Z. Xu X. (2016). Gut microbiome remodeling induces depressive-like behaviors through a pathway mediated by the host’s metabolism. Mol. Psychiatry 21 (6), 786–796. 10.1038/mp.2016.44 27067014

[B145] Zhou Z. Meng M. Ni H. (2017). Chemosensitizing effect of astragalus polysaccharides on nasopharyngeal carcinoma cells by inducing apoptosis and modulating expression of Bax/Bcl-2 ratio and caspases. Med. Sci. Monit. 462–469. 10.12659/msm.90317023 28124680 PMC5291085

[B45] Zhu B. Zhai Y. Ji M. Wei Y. Wu J. Xue W. (2020). Alisma orientalis beverage treats atherosclerosis by regulating gut microbiota in ApoE(-/-) mice. Front. Pharmacol. 11, 570555. 10.3389/fphar.2020.570555 33101028 PMC7545905

