# Supplementary figures and images for: Jianpi Jieyu Decoction, An Empirical Herbal Formula, Exerts Psychotropic Effects in Association With Modulation of Gut Microbial Diversity and GABA Activity
Source: Front Pharmacol. 2021 Apr 14;12:645638. doi: 10.3389/fphar.2021.645638 (PMC8079981; doi:10.3389/fphar.2021.645638)

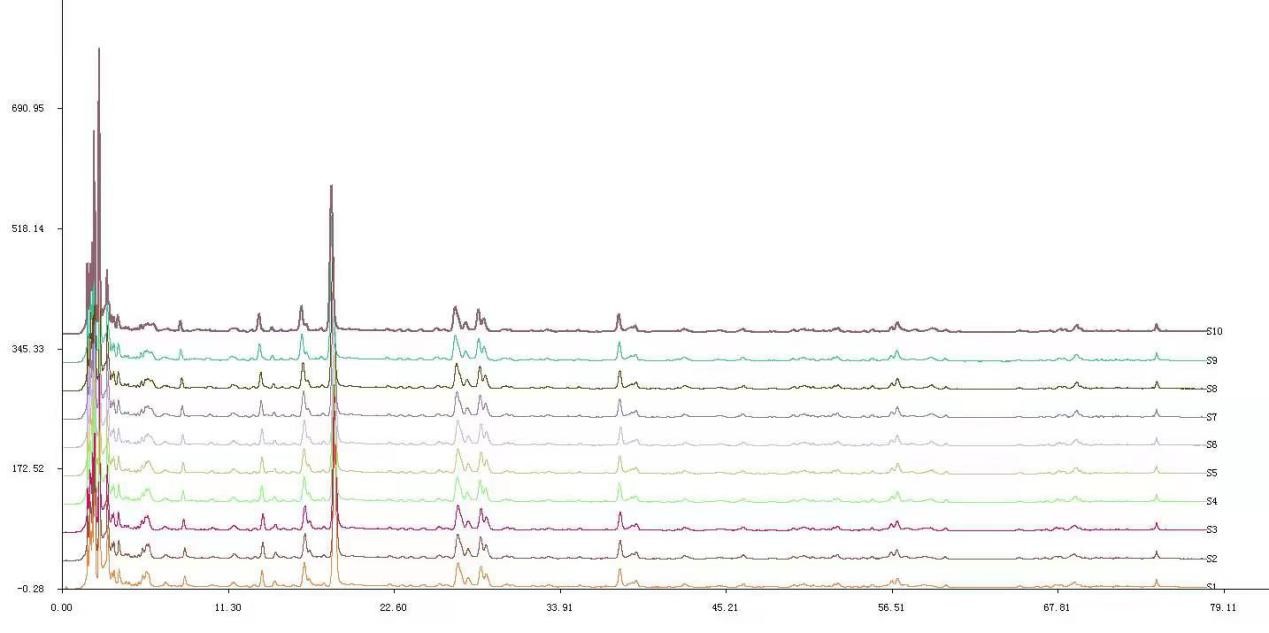

Supplement: Supplementary file 1 [file image1.tiff]
